# Parental perception of child’s weight status and subsequent BMIz change: the KOALA birth cohort study

**DOI:** 10.1186/1471-2458-14-291

**Published:** 2014-03-31

**Authors:** Sanne MPL Gerards, Jessica S Gubbels, Pieter C Dagnelie, Stef PJ Kremers, Annette Stafleu, Nanne K de Vries, Carel Thijs

**Affiliations:** 1 Department of Health Promotion, and NUTRIM School for Nutrition, Toxicology and Metabolism, Maastricht University, Maastricht, The Netherlands; 2 Department of Epidemiology, Maastricht University, Maastricht, The Netherlands; 3 CAPHRI School of Public Health and Primary Care, Maastricht University, Maastricht, The Netherlands; 4 TNO, Zeist, The Netherlands

**Keywords:** Body Mass Index, Children, Overweight, Parents, Weight Perception

## Abstract

**Background:**

Parents often fail to correctly perceive their children’s weight status, but no studies have examined the association between parental weight status perception and longitudinal BMIz change (BMI standardized to a reference population) at *various* ages. We investigated whether parents are able to accurately perceive their child’s weight status at age 5. We also investigated predictors of accurate weight status perception. Finally, we investigated the predictive value of accurate weight status perception in explaining children’s longitudinal weight development up to the age of 9, in children who were overweight at the age of 5.

**Methods:**

We used longitudinal data from the KOALA Birth Cohort Study. At the child’s age of 5 years, parents filled out a questionnaire regarding child and parent characteristics and their perception of their child’s weight status. We calculated the children’s actual weight status from parental reports of weight and height at ages 2, 5, 6, 7, 8, and 9 years. Regression analyses were used to identify factors predicting which parents accurately perceived their child’s weight status. Finally, regression analyses were used to predict subsequent longitudinal BMIz change in overweight children.

**Results:**

Eighty-five percent of the parents of overweight children underestimated their child’s weight status at age 5. The child’s BMIz at age 2 and 5 were significant positive predictors of accurate weight status perception (vs. underestimation) in normal weight and overweight children. Accurate weight status perception was a predictor of higher future BMI in overweight children, corrected for actual BMI at baseline.

**Conclusions:**

Children of parents who accurately perceived their child’s weight status had a higher BMI over time, probably making it easier for parents to correctly perceive their child’s overweight. Parental awareness of the child’s overweight as such may not be sufficient for subsequent weight management by the parents, implying that parents who recognize their child’s overweight may not be able or willing to adequately manage the overweight.

## Background

Childhood overweight and obesity are rapidly increasing public health problems [1]. Preventive interventions are increasingly being aimed at parents of overweight children, [2] who are responsible for a substantial part of their children’s nutrition and physical activity behaviours. However, parents often fail to recognize their child as overweight [3,4]: a systematic review on parents’ perceptions of their children’s weight status showed that more than half of the parents of overweight children underestimate their child’s overweight, perceiving their child as normal weight [5].

Several studies have examined cross-sectional factors related to accurate parental perception of their child’s weight status: lower child birth weight [6], a higher physical activity level of the child [7], having a female child [7,8], higher age of the child [9], higher parental educational level [7,10,11], higher maternal health literacy level [12], parents being a first-generation immigrant (vs. second-generation) [10] and higher maternal age [10], have been found to be associated with accurate parental perception of their child’s weight status. Findings regarding the relationship between parental weight perception and children’s actual BMI have been inconsistent: some studies found a positive association between children’s BMIz (BMI standardized to a reference population) and accurate weight status perception in overweight children, [7,9] whereas one study found that children’s BMIz were positively associated with underestimation of the children’s overweight status [13]. The relationship between the mother’s weight status and perception of their children’s weight is also still unclear. Some studies showed that higher maternal BMI was associated with underestimation of their children’s weight status [6,14,15], while another study found that mothers with a lower BMI were more likely to underestimate their children’s overweight [13]. Discrepant findings may be due to different populations, sampling methods and/or definitions of overweight [7,9].

It is also important to explore the association between parental weight status perception and subsequent weight change of the child, as this provides an indication of whether parents are able to accurately manage their child’s weight. Kroke et al. [16] have performed such a longitudinal investigation into the relation between mothers’ perception of the weight status of their children at different ages on the one side, and the children’s actual weight at age 7 on the other side. They compared the BMI change in children whose mothers correctly perceived their child’s weight status with that in children whose mothers did not. They showed that BMIz change was only significantly different between the two groups of mothers for the 6-months-old children: maternal misperception at 6 months was associated with unfavourable subsequent weight development up to age 7. However, the sample sizes of the different subgroups in this study were relatively small, hampering inferences from this study.

No earlier studies have taken account of the child’s prior BMI in predicting parental weight status perception. The aims of the current study were to explore whether parents are able to accurately perceive their child’s weight status (around age 5); to examine predictors of accurate parental weight status perception, including children’s prior BMI at 2 years as a predictor; and to investigate children’s longitudinal subsequent BMIz change up to age 9 years in relation to parental perceptions or misperceptions of their child’s weight status.

## Methods

### Respondents and procedure

The KOALA Birth Cohort Study is a prospective cohort study in the Netherlands which started in 2000. KOALA is a Dutch acronym for Child, Parent, and health: Lifestyle and Genetic constitution. Pregnant women were recruited from an existing cohort from a study of pregnancy-related pelvic girdle pain (N = 2343; regular group). Additional participants were recruited among ‘alternative lifestyle’ circles (e.g., organic food shops, anthroposophist midwives) [17]. This latter group of women could have an alternative lifestyle as regards aspects like child rearing, dietary habits, vaccination schemes or antibiotics use (N = 491; alternative group).

The Medical Ethics Committee of the University Hospital and Maastricht University approved the study. All participants signed an informed consent form. A total of 2834 mothers participated and completed questionnaires during pregnancy as well as at regular intervals after birth.

### Questionnaire

In the current study, we analysed child and parental characteristics by means of a questionnaire which parents completed when the children were approximately 5 years old (mean 5.01 ± 0.53). Parents were instructed to fill out the questionnaire in collaboration. The total number of questionnaires returned was 2066 (73% of 2834). In addition, we analysed parental reports of the weight and height of their children at the ages of around 2 (mean 1.83 ± 0.32; N = 1735; 84% of the 2066 respondents at age 5), 5 (mean 5.01 ± 0.53; N = 1915; 93%), 6 (mean 6.48 ± 0.61; N = 1475; 71%), 7 (mean 7.19 ± 0.65; N = 1362; 66%), 8 (mean 8.21 ± 0.64; N = 1288; 62%) and 9 (mean 9.16 ± 0.64; N = 1394; 67%). The weight and height of a substantial proportion of the children (N = 372) at the age of 5 were measured by trained research assistants [18].

### Weight status perception

Parental perception of their child’s weight status was assessed around age 5 by the following questions: ‘How would you describe your child’s weight currently?’. Answer categories were: clearly underweight, underweight, normal weight, overweight and clearly overweight. In view of the very small proportions of children classified in the two extreme categories, we recoded the answers into three categories: underweight (including both clearly underweight and underweight), normal weight and overweight (including both overweight and clearly overweight).

### Parental characteristics

At the child’s age of 5, both parents were asked to report their own weight and height, which were used to calculate their body mass index (BMI; weight (kg)/(height (m))^2^). A BMI below 18.5 kg/m^2^ was regarded as underweight, and a BMI above 25 kg/m^2^ as overweight. Additionally, parents were asked to indicate the number of hours they worked per week, their country of birth and their highest completed level of education. Country of birth was recoded into Netherlands vs. other. Education was categorized into three levels: low, medium and high, based on international classification systems [19] (low: primary school, lower vocational education and lower general secondary education; medium: intermediate vocational education, higher general secondary education and university preparatory education; high: higher vocational education and university). From previous questionnaires, we derived the mother’s age at the birth of the child and the recruitment channel (alternative vs. regular).

### Child characteristics

The child’s date of birth and gender were derived from previous questionnaires. BMI was recoded into BMIz standardized for age and gender, compared to the national reference population (i.e., the Fourth Dutch National Growth Study) [20]. Weight status was recoded into three categories, based on BMIz: underweight (<5th percentile, BMIz = 1.04), normal weight (5th – 84th percentile, BMIz 1.05 – 1.64) and overweight (≥85th percentile, BMIz = 1.65) [21].

### Data analyses

All statistical analyses were conducted using SPSS 19.0. P-values <0.05 were considered statistically significant. We performed descriptive analyses (means and frequencies) to summarize child and parent background characteristics. Crosstabs were used to compare the distribution of parents who accurately perceived their child’s weight and those who did not at age 5 years, for children of different weight categories. Children who were currently underweight were excluded from further analyses, because they were not our target population.

### Predictors of accurate weight status perception

Multiple backward binary logistic regressions were conducted to predict which parents correctly estimated their child’s weight status cross-sectionally at age 5 (1 = accurate perception of child’s weight status, 0 = underestimation of child’s weight status), using child characteristics (BMIz at age 2, BMIz at age 5, gender) as well as parental characteristics (BMI of both parents, age of mother at birth of child, educational level of both parents, country of birth of both parents, employment status of both parents (hours per week) and recruitment channel) as predictors. This was done separately for children with normal weight and overweight at the age of 5. First, the predictors were entered one by one to examine the bivariate correlations. Second, all predictors were entered simultaneously and were then backward-deleted from the model based on their significance level, starting with the predictor with the highest P-value, until only statistically significant predictors were left, in order to adjust for possible confounding. Third, we performed separate analyses including an interaction term between measurement method (weight and height by parental report vs. measured by a trained research assistant) and BMIz in the models, to check whether the results were influenced by the measurement method used for weight and height. In none of the models a significant interaction effect was found between measurement method (self-report vs. measured) and BMIz (P-values all > 0.05). We also evaluated whether the results differed between the regular recruitment channel and alternative recruitment channel by adding an interaction term in the model between recruitment channel and BMIz in the models. None of these interaction terms were statistically significant.

### Longitudinal BMIz change

Multivariate backward linear regression analyses were performed for the children who were overweight at age 5, to examine the relationship between accurate parental classification of their child’s weight status at age 5 on the one hand, and subsequent changes in the child’s BMIz over time on the other (from age 5 up to ages 6, 7, 8 and 9 years, respectively). The following covariates were included in the basic model: gender of the child, BMI of both parents, educational level of both parents, country of birth of both parents, employment status of both parents (hours per week) and recruitment channel. Again, the predictors were backward-deleted from the model until only statistically significant predictors were left. We included an interaction term between measurement method (self-report vs. measured) and accurate weight status perception in the analysis to investigate whether we should conduct separate analyses for children whose weight and height were measured and children whose weight and height were self-reported by their parents. In none of the models was a significant interaction effect found between measurement method and accurate weight status perception (P-values all >0.05).

Finally, we entered an interaction term between weight status perception and BMIz at age 5 into these regression models, to explore whether BMIz change was significantly different for children whose parents accurately estimated their child’s weight status as compared to children whose parents underestimated their weight status. The robustness of the analyses was examined by varying the inclusion and coding of the covariates.

## Results

### Study population

Table 1 lists the general characteristics of the children and parents for whom both questionnaire data and BMIz were available at age 5. At that age, 83% of the children had a normal weight and 9% were overweight (or obese). Almost all parents had been born in Netherlands, and 54% had a high educational level. Thirty-two percent of the mothers and 46% of the fathers were overweight or obese. Attrition at 6, 7, 8 and 9 years was non-selective with regard to BMIz and parental perception of their child’s weight status at 5 years.

**Table 1:** Demographics and weight-related characteristics of children and parents of the KOALA cohort around age 5 (N = 1915)

		**Mean ± sd**	**Prevalence**
Child	Gender		Male: 51%
			Female: 49%
	Age	5.01 ± 0.53	
	BMI z-score	−0.27 ± 0.99	
	Weight status		Underweight: 9%
			Normal weight: 83%
			Overweight + obesity: 9%
Mother	Age at birth of child	32.21 ± 3.78	
	Country of birth		Netherlands: 97%
			Other: 3%
	Educational level		High: 54%
			Medium: 38%
			Low: 8%
	Employment (*hours per week*)	17.87 ± 10.93	
	Alternative lifestyle		18%
	BMI	23.94 ± 3.79	
	Weight status		Underweight: 2%
			Normal weight: 67%
			Overweight + obesity: 32%
Father	Country of birth		Netherlands: 96%
			Other: 4%
	Educational level		High: 53%
			Medium: 34%
			Low: 13%
	Employment (*hours per week*)	37.89 ± 9.94	
	BMI	25.07 ± 3.09	
	Weight status		Underweight: 1%
			Normal weight: 53%
			Overweight + obesity: 46%

### Weight status perception

Table 2 present the distribution of parental perceptions of their child’s weight status and the child’s actual weight status at the age 5. A large majority (93%) of the parents perceived their child’s weight status at 5 years to be normal. Even parents of overweight and underweight children mainly perceived their child as having a normal weight. A substantial proportion (78%) of the parents of underweight children *over*estimated their child’s weight status, whereas 85% of the parents of overweight children *under*estimated the weight status of their child.

**Table 2:** Parental cross-sectional perception of child’s weight status at age 5 years by the child’s actual weight status at age 5 years

**Parental perception of child’s weight status**	**Actual weight status of child**			**Total**
	**Underweight**	**Normal weight**	**Overweight**	
Underweight	**36 (22%)**	67 (4%)	2 (1%)	105 (6%)
Normal weight	125 (78%)	**1504 (96%)**	141 (83%)	1770 (93%)
Overweight	0	3 (0%)	**26 (15%)**	29 (2%)
Total	161 (100%)	1574 (100%)	169 (100%)	1904^a^

*Accurate weight perception in total sample* = *82*% (*see bold numbers in the Table*).

^a^N deviates from total sample because of missing values.

### Predictors of accurate weight status perception

Bivariate analyses showed that accurate parental perception was significantly associated with BMIz at 2 years (OR = 0.817, P = 0.09) and BMIz at 5 years (OR = 15.322, P = 0.00) for overweight children and BMIz at 2 years (OR = 2.780, P = 0.00) and BMIz at 5 years (OR = 4.458, P = 0.00) for normal weight children. Table 3 presents the adjusted predictors of accurate parental perception of their child’s weight status at age 5. BMIz at ages 2 and 5 in both the overweight and normal weight sample were significant predictors of accurate weight status perception. Compared to children with lower BMIz at 5 years, parents of children with higher BMIz were more likely to correctly perceive their child as normal weight or overweight. In addition, parents of children whose BMIz was higher at 2 years were more likely to accurately perceive their child’s weight status at 5 years than parents whose child had a lower BMIz at the age of 2. In normal weight children, a high paternal educational level was a significant predictor of accurate perception of the child’s weight status. None of the other demographic variables showed a significant association with the accuracy of the weight status perception.

**Table 3:** Odds ratios for predictors of accurate parental perception of child’s weight status at 5 years

	**Accurate parental weight status perception**^**a**^	
	**Odds ratio (95% CI)**	
	**Normal weight children**	**Overweight children**
	***N*** **=** ***1369***	***N*** **=** ***146***
BMIz at 2 years	2.39*** (1.72; 3.32)	2.88*** (1.58; 5.26)
BMIz at 5 years	2.92*** (1.72; 4.95)	13.86*** (3.77; 50.93)
Educational level of father		
High vs medium	1.83* (1.00; 3.35)	- ^*b*^
Low vs medium	1.53 (0.62; 3.74)	- ^*b*^

*P ≤ 0.05; **P ≤ 0.01;***P ≤ 0.001.

Results of multivariate backwards binary logistic regression analyses. Non-significant (P >0.05) predictors were backward-deleted. Variables that were excluded from all models due to non-significance were child’s gender, BMI of both parents, recruitment channel, educational level of the mother, country of birth of both parents, employment status of both parents and age of mother at birth of child.

^a^1 = accurate perception of child’s weight status, 0 = underestimation of child’s weight status.

^b^Variable not included in the final backwards regression model (n.s.).

### Longitudinal BMIz change of 5-year-old overweight children

We predicted the changes in BMIz from age 5 to ages 6, 7, 8 and 9 in overweight children (see Table 4). Significant predictors of changes in BMIz in all four models were accurate weight status perception at age 5 and BMIz at age 5. Accurate weight status perception at 5 years was associated with a greater increase in BMIz from 5 years to 6, 7, 8 and 9 years, compared to underestimation of the child’s weight status. In addition, BMIz at 5 years was negatively associated with changes in BMIz from age 5 to ages 6, 7, 8 and 9 years.

**Table 4:** Predictors of change in child’s BMIz from age 5 up to age 9 for children who were overweight at age 5

	**Unstandardized regression coefficients B (95% CI)**			
	*6 years*	*7 years*	*8 years*	*9 years*
	*N* = *129*	*N* = *110*	*N* = *103*	*N* = *102*
Accurate perception of child’s weight status (*0* = *underestimation*, *1* = *accurate*)	0.70***	0.96***	0.89***	0.99***
	(0.31;1.08)	(0.56;1.36)	(0.41;1.37)	(0.48;1.49)
Child BMIz at 5 years	−0.57**	−0.97***	−0.81***	−0.84***
	(−0.94;-0.21)	(−1.34;-0.60)	(−1.27;-0.37)	(−1.33;-0.35)
Total variance explained by the model (R^2^)	0.18	0.29	0.22	0.24

*P ≤0.05; **P ≤0.01;***P ≤0.001.

Results of multivariate backwards linear regression analyses. Non-significant (P >0.05) predictors were backward-deleted. Variables that were excluded from all models due to non-significance were gender of the child, recruitment channel, country of birth of both parents, employment status of both parents, mother’s BMI and age of mother at the birth of the child.

Analyses were controlled for significant background characteristics: educational level of father, educational level of mother and BMI of father.

**Figure 1:**
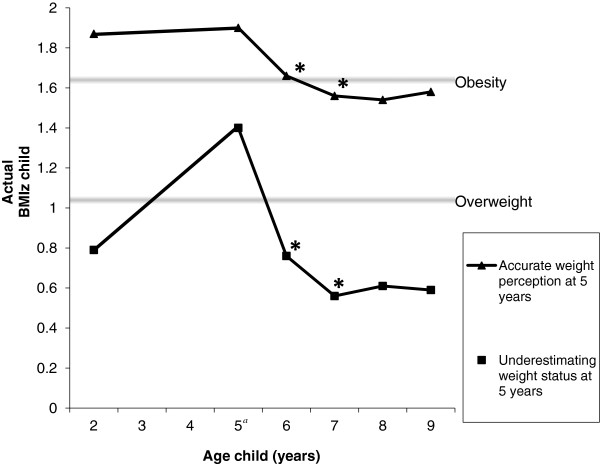
**BMI development of overweight children whose parents accurately perceived or underestimated their child’s weight status at the age of 5. **^*a*^Weight status perception was assessed at the child’s age of 5; cut-off points for overweight and obesity were ≥85th percentile (BMIz = 1.04) and ≥95th percentile (BMIz = 1.65), respectively. *Significantly different patterns between the two groups in BMI change from age 5 to ages 6 and 7.

We found a significant interaction between BMIz at age 5 and the perception of a child’s weight status (accurate vs inaccurate) in terms of predicting the child’s change in weight from age 5 to ages 6 and 7 (β = 1.116; P = 0.030 and β = 1.608; P = 0.004, respectively). Figure 1 shows the longitudinal change in BMIz (based on means) of the overweight children whose parents underestimated their weight status and of the children whose parents correctly perceived their weight status at age 5. Children whose parents accurately perceived their weight status had a higher BMIz at 5 years and were more likely to maintain this higher weight, whereas children whose parents underestimated their weight had only one peak in their weight, at age 5 years.

## Discussion

This study has yielded information about the longitudinal change of children’s BMI in relation to parental perception of their child’s weight status. We found that accurate weight status perception at age 5 years was significantly associated with higher BMI in overweight children until the age of 9 years (4 years follow-up), corrected for actual BMIz at age 5 years. Furthermore, children of parents who correctly perceived their child as being overweight showed a consistently higher BMI at different ages, compared to children of parents who underestimated their child’s overweight. A consistently higher BMI probably made it easier for parents to correctly perceive their child’s overweight [6]. Nevertheless, even after correction for actual BMIz, the association between accurate weight status perception and higher BMI at follow-up remained statistically significant. This would imply that parental awareness of the child’s overweight as such is not protective against subsequent overweight development. This contradicts the findings of Kroke and colleagues [16] who reported that maternal misperception about overweight children aged 6 months had an unfavourable effect on the child’s weight development, compared to mothers who perceived their child’s weight status correctly. However, as the researchers themselves acknowledged, [16] determination of actual weight status using BMI in children below the age of 2 years may be an inappropriate measure, [22] and the feeding mode (breastfeeding or bottle-feeding) had a differential effect on early body mass development [23]. Also, their study included relatively small subsamples, impeding external validity.

Eighty-five percent of the parents of overweight children in our study underestimated their child’s weight status. Similar percentages were found in previous studies, with 72 - 90% of the parents underestimating the weight status of their overweight child [4,8,10,11,13]. Several potential causes may underlie inaccurate parental weight status perception [9]. First, parents may not recognize their child as overweight; they just do not see it or do not have the skills to see it. Another cause may be that parents do not understand what overweight means. This is suggested by the findings of Jain et al. [24] who performed qualitative interviews with low-income mothers to understand their perceptions of when a child is overweight. The authors found that these mothers did not accept the classifications used by health professionals to determine a child’s overweight. Finally, parents may feel reluctant to admit that their child is overweight [25]. Other studies reported that parental perception of their child’s weight status was related to the mother’s own overweight status, which may influence their norm regarding what is a normal weight [6,14]. Although a relatively large proportion of the parents in the current study were overweight or obese, we could not find such an association between parental BMI and the parent’s perception of their child’s weight status or the child’s BMIz change.

Predictors of accurate weight status perception, in both overweight and normal weight children aged 5 years, were the children’s actual BMIz at 5 years and the children’s BMIz at 2 years (both predictors being positively associated with accurate weight status perception). Other studies have also shown a positive association between actual cross-sectional BMI(z) and weight status perception, [7,9] but we are the first to investigate the influence of prior BMI on the parental perception of previous body weight status. In normal weight children, high educational level of the father was an additional significant predictor of accurate perception. In line with this, previous studies [7,10,11] found that mothers with a low educational level were more likely to underestimate their child’s weigh status. We did not find any evidence that other demographic variables are significant predictors of accurate weight perception, which appears to contradict what other studies have reported [7-11,13]. An explanation for the discrepancies between studies may be the use of different instruments for measuring weight status perception. Some studies (including the current study) have used written questionnaires to assess parental perception of their child’s weight status, [9-14] whereas others have used an interview technique, [6,7,16] which may make it even more emotionally challenging for parents to admit that their child is overweight compared to an anonymous questionnaire [26]. Another possible cause of discrepancies between study results may be differences in general characteristics of the study populations between earlier studies and ours. The KOALA cohort is relatively healthy; e.g., 9% of the 5-year-old children were overweight or obese, compared to 13% and 18% of 5-year-old boys and girls in the general Dutch population the Dutch boys of the Dutch girls at the age of [27] The cohort also included relatively highly educated parents: 53% of the parents had a high education level, compared to 26-31% of the general Dutch population [28]. These specific general characteristics of the KOALA cohort may mean that the influence of several predictors was not detected, because of power issues.

In the present study, 5-year-old children whose parents underestimated their child’s overweight status were generally only overweight at that particular measurement, whereas the children whose parents accurately perceived their child’s overweight consistently had a higher average BMI over time. This indicates that children whose parents accurately perceive their weight differ in weight status from the children whose parents underestimate their weight status. We found a similar pattern for parents of normal weight children: children of parents, who correctly perceived their child as normal weight, had a consistently higher BMI compared to children of parents who estimated their child as underweight. This implies that one should be very careful when examining parental perceptions of the child’s weight status in only one cross-sectional weight measurement. It seems that parents can accurately take into account their child’s weight history in assessing his or her weight status at one particular point in time. Alternatively, this phenomenon may be a statistical artefact, caused by measurement errors in either the child’s body weight or parental weight status perception, or regression to the mean.

Accurate weight status perception may be an important prerequisite for involving parents in childhood obesity interventions [25]. In general, parents who are not aware that their child is overweight, will probably not feel the need to become involved in such an intervention [29,30]. In one adolescent family-based intervention, for example, parental weight status perception proved an important predictor of treatment initiation [31]. Nevertheless, accurate weight status perception alone is not sufficient. Parents may not have sufficient motivation, skills or parenting practices to manage their child’s overweight, or they may even use counterproductive strategies, such as extreme restriction or overcontrolling of unhealthy energy balance-related behaviours, to address their child’s overweight [32]. This may result in further BMI increases in the long run. Parents therefore need to be enabled to develop adequate skills to manage their child’s overweight in family-based interventions [33,34].

Some limitations should be taken into account when interpreting our results. Almost all data were self-reported by parents, which may have led to bias. However, the weight and height data at age 5 were partly measured by trained research assistants. We tested whether the self-report and measured data led to different results, using interaction terms in the models, and found no difference between the two measurement methods. This is line with the findings by Scholtens et al. [35] who reported that parental reports of their child’s actual weight and height are relatively valid. However, parents of overweight children tend to underreport their child’s weight [35,36], indicating that even more parents underestimate their child’s weight status than we could establish in the current study. Unfortunately, we were not able to conduct separate analyses of parental weight status perception for the group of obese children, as the small number of children in this subsample (N = 32) mean there was insufficient statistical power for such analyses. Furthermore, we did not collect any data on parental behaviours, which was beyond the scope of our study. An additional limitation regards the representativeness of the KOALA cohort, as already noted. Another disadvantage of the KOALA cohort might be completing questionnaires on a regular basis, which may increase parental attention for the questioned topics and may influence their behaviour.

To our knowledge, this is the first study to examine parents’ perceptions of their child’s weight status in relation to longitudinal BMIz change, both prior and subsequent to the assessment of parental perception. It would be valuable to further investigate the effect of underestimation on children’s weight status, specifically with regard to causality and underlying mechanisms explaining the associations found in the current study. Future studies with a larger sample of overweight children should investigate what differences there are, within the group of ‘consistently’ overweight children, between parents who accurately estimate their child’s weight status and those who underestimate it.

## Conclusions

A relatively large proportion of the parents misperceive their child’s weight status, especially parents of children with a deviant weight status (underweight or overweight). The most important predictors of accurate parental weight perception are the children’s current BMI and prior BMI. This indicates that children whose parents accurately perceive their weight differ in weight status from the children whose parents underestimate their weight status. In addition, children whose parents accurately perceive their overweight child as being overweight showed a greater increase in BMI over time. Mere awareness of the child’s weight status thus seems to be unfavourably associated with the child’s BMIz change. It would therefore appear appropriate to develop and test new interventions in which parents are taught skills for managing their children’s weight-related behaviours.

## Competing interest

The authors declare that they have no conflict of interest.

## Authors’ contributions

SG was the primary investigator in this study and wrote the first draft of the paper. JG, PC, SK, AS, NK and CT were involved in revising the manuscript. All authors read and approved the final manuscript.

## Pre-publication history

The pre-publication history for this paper can be accessed here:

http://www.biomedcentral.com/1471-2458/14/291/prepub
